# Schema: A Quantified Learning Solution to Augment, Assess, and Analyze Learning in Medicine

**DOI:** 10.7759/cureus.81803

**Published:** 2025-04-06

**Authors:** Deepu Sebin, Vishwin Doda, Skanthavelan Balamani

**Affiliations:** 1 Internal Medicine, Neuroglia Health Private Limited, Bengaluru, IND; 2 Education, Neuroglia Health Private Limited, Bengaluru, IND; 3 Medicine and Surgery, Jawaharlal Nehru Medical College, Wardha, IND

**Keywords:** curriculum integration, e-learning, innovation in medical education, quantified learning, technology enhanced education

## Abstract

Quantified learning is the use of digital technologies, such as mobile applications, cloud-based analytics, machine learning algorithms, and real-time performance tracking systems, to deliver more granular, personalized, and measurable educational experiences and outcomes. These principles, along with horizontal and vertical integrative learning, form the basis of modern learning methods. As we witness a global shift from traditional learning to competency-based education, educators agree that there is a need to promote quantified learning. The increased accessibility of technology in educational institutions has allowed unprecedented innovation in learning. The convergence of mobile computing, cloud computing, and Web 2.0 tools has made such models more practical. Despite this, little has been achieved in medical education, where quantified learning and technology aids are limited to a few institutions and used mainly in simulated classroom environments. This innovation report describes the development, dynamics, and scope of Schema, an app-based e-learning solution designed for undergraduate medical students to promote quantified, integrative, high-yield, and self-directed learning along with feedback-based self-assessment and progress monitoring. Schema is linked to a database of preclinical, paraclinical, and clinical multiple choice questions (MCQs) that it organizes into granular subtopics independent of the core subject. It also monitors the progress and performance of the learner as they solve these MCQs and converts that information into quantifiable visual feedback for the learners, which is used to target, improve, revise, and assess their competency. This is important considering the new generation of medical students open to introducing themselves to technology, novel study techniques, and resources outside the traditional learning environment of a medical school. Schema was made available to medical students as part of an e-learning platform in 2022 to aid their learning. In addition, we also aim to use Schema and the range of possibilities it offers to gain deeper insights into the way we learn medicine.

## Introduction

According to the WHO, more than 90,000 students were admitted to medical colleges in India, and 1.36 million worldwide in 2022. These students undertake the task of learning the nuances of medicine and transforming into competent medical professionals over the course of their undergraduate education. The students from Generation Z (born between 1997 and 2012) are the first cohort to have had access to modern technology during their undergraduate years, with ready access to the Internet. As a result, they are open to new learning techniques and technology and expect a personalized approach to medical learning [1]. E-learning, virtual learning environments, and simulated clinical experiences have inspired the need for a quantified, self-directed, and integrated approach to learning and assessing competencies in medicine. With changing paradigms, the learning systems in medical education must also evolve to cater efficiently and holistically to this new generation of learners. The opportunity to do so is evident in the fact that most medical students report using mobile devices to aid their learning in domiciliary and clinical settings more than ever before [2]. While methodologies such as problem-based learning (PBL) and team-based learning (TBL) have been incorporated into medical curricula since the 1970s, many institutions, particularly in developing regions, continue to rely heavily on subject-specific reading and subjective evaluations. However, a global trend toward competency-based education is accelerating the shift toward more integrated, technology-aided, and objective assessment models. Perhaps the single most crucial shift in modern-day medical education is the growing role of mobile apps, which have been shown to aid learning and improve performance in solving clinical problems [3-5]. This innovation report aims to describe Schema, an app-based solution, along with its development, dynamics, potential use cases, and scope. It also explores the application of Schema to recognize en masse trends in the competency of medical students in various topics of the curriculum by observing the percentage correctness of the responses to each multiple choice question (MCQ).

## Technical report

Approach

Schema is a first-of-its-kind learning tool designed to promote self-directed, integrated, and quantified learning of high-yield subtopics with active feedback to the learner. This innovative approach has refined and granularized the core undergraduate medical curriculum into 1,947 "schemas" or sub-topics, which are easily navigable through our user interface. The development of Schema was driven by the need to categorize a set of 16,000 preclinical, paraclinical, and clinical Single Best Answer Multiple Choice Questions (SOC-MCQs) from 19 medical subjects, including Anatomy, Physiology, Biochemistry, Microbiology, Pathology, Pharmacology, Forensic Medicine, Ophthalmology, Otorhinolaryngology, Community Medicine, Medicine, Surgery, Obstetrics and Gynaecology, Pediatrics, Dermatology, Psychiatry, Radiology, Anaesthesiology, and Orthopedics into a more granular format for easier identification, navigation, and integration.

An ideal integrative system for quantified learning should satisfy certain parameters. It should granularize the medical curriculum into highly specific subtopics and be subject-agnostic to allow for seamless horizontal and vertical integration. Additionally, it should be easily navigable to facilitate access and have a definite organizational structure. The system should also be capable of providing feedback on the extent to which a learner has completed the curriculum and offer positive feedback regarding the performance of learners in each topic. Importantly, it should highlight high-yield topics so that the learner can maximize the benefits from their studies and not act as a shortcut to completing studies but rather as an augmentation to the learner’s efforts for efficient learning.

Curricular organization, integration, and alignment

**Figure 1:**
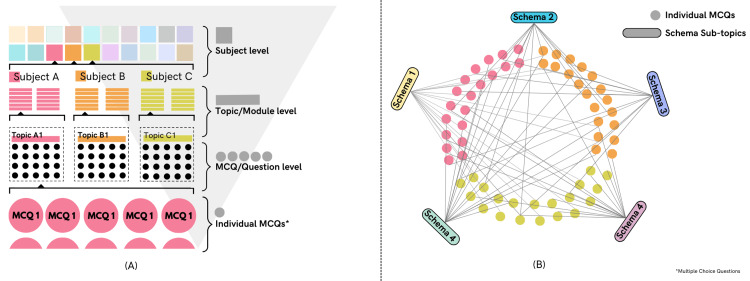
Systems of curricular organization: (A) traditional, (B) Schema model of integrative organization. MCQ: Multiple choice question.

Unlike the traditional models that organize the curriculum into a subject-specific hierarchy, we consolidated MCQs according to Schema subtopics, irrespective of the subject they would traditionally be categorized in. In other words, Schema is subject agnostic. Every MCQ is linked to all Schema subtopics related to it, and each Schema subtopic is linked to relevant MCQs that fall within its scope, in a non-exclusive manner as shown in Figure 1. This approach is novel in the domain of learning, with the resulting outcome being seamless vertical and horizontal integration while maintaining some boundaries within the content, thus removing redundancy and providing interconnectedness. This quasi-segregated model of curricular organization enables the use of Schema for a targeted approach in teaching, self-directed learning, assessment, and pinpointing knowledge gaps. Organizing the MCQs into their respective Schema subtopics was a challenging task. First, all the subtopics that each MCQ could be linked to were listed. The list of such subtopics was then compiled to create a central database containing all subtopics representing a granular subtopic independent of a subject or chapter. Some examples of these subtopics are “Ivermectin,” “Anaphylaxis,” and “Kernicterus.” Some subtopics were granularized into more specific subtopics, often when different aspects of a topic, disease, or condition were. For example, the subtopic Diabetes Mellitus was granularized into “Diabetes Mellitus: Pathophysiology and Clinical Features,” “Diabetes Mellitus: Investigations,” “Diabetes Mellitus: Treatment,” and “Diabetes Mellitus: Macrovascular and Non-vascular Complications.” It is to be noted that a significant number of MCQs were linked to more than one Schema topic, resulting in nesting integration across horizontal and vertical domains. At the end of this exercise, a total of 3010 such subtopics were identified, which nearly encompassed the entire medical curriculum in a granular format.

The next exercise was to manually identify and label high-yield subtopics based on their cruciality in the clinical practice of a physician, and the changing paradigms of globally relevant diseases. A total of 1,947 such high-yield topics were recognized and compiled to form the Schema list of high-yield subtopics. As opposed to automated approaches such as text-mining, a manual approach was selected for identification and labeling of subtopics. Text-mining-based approaches may have several known drawbacks such as artifacts, lack of contextual understanding, and inability to consider semantic ambiguity or variability in terminology. Technology-based or automated detection of such overlaps has also been previously attempted using text mining techniques and has been shown to have several drawbacks [6-7]. The manual approach of categorizing each of the thousands of MCQs and analyzing overlaps is unique, avoids these drawbacks, and offers the additional advantage of a more insight-based identification of relevant subtopic tags. The manual approach helped us map overlaps within the curriculum of the same disease, anatomical structure, or conditions assessed under different subjects due to the different approaches practiced under different specializations, or are assessed on different occasions in the teaching timeline resulting in duplication of effort and reduced integrated learning.

The Schema interface

The interface of Schema serves the integral function of aiding navigation within the Schema by visually bringing together the extensive Schema database and presenting it to the learner in a user-friendly manner. The Schema interface serves the following functions:

Intuitively Navigate, Sort, and Analyze the Schema List of Topics

**Figure 2:**
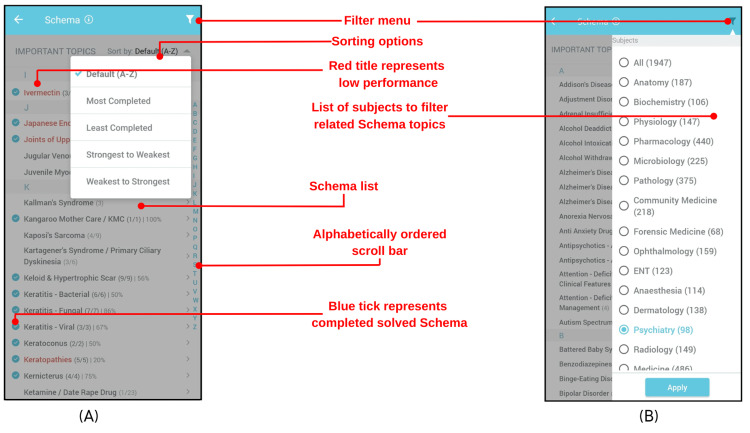
Schema list interface depicting functions and feedback.

In order to make the navigation within the Schema intuitive for learners, a simple yet functional interface was desirable. It must provide the learner with intuitive navigation through Schema. The alphabetical sorting bar allows intuitive navigation to a specific Schema entry. The list of Schema topics can also be sorted alphabetically, from most to least completed and vice versa and from strongest to weakest and vice versa as shown in Figure 2A. This allows the learner to analyze their progress, focus efforts to cover the curriculum, focus on areas that need improvement, and further improve their strengths. The filter menu allows the learner to focus on Schema topics in which MCQs from that particular subject occur, as shown in Figure 2B.

Tapping on a Schema title reveals the list of MCQs linked to it, organized by the module they originally covered and the date the module was last attempted. An option to bookmark any MCQ that the learner finds important or difficult is also provided, which facilitates review.

Provide Feedback About the Learner’s Progress and Performance

The Schema interface contains several elements that provide users with feedback on their progress and performance in both visual and quantified forms.

In the Schema list interface, these elements include:

Blue ticks: A blue tick conveys to the learner that all MCQs in a Schema topic have been attempted. Collectively, blue ticks allow the learner to perceive progress and the extent of curricular coverage.

Red titles: Red titles indicate a low average percent score (<50%) in a Schema topic. This provides the learner with feedback on which Schema topics need improvement.

Within an individual Schema topic, feedback elements include:

Solved MCQ count: It quantifies the number of MCQs solved by the learner out of the total MCQs in a given Schema topic. Furthermore, it quantifies the number of correct, wrong, and skipped MCQs, providing the learner with insight regarding their overall performance in a particular Schema topic.

Score: The percentage of MCQs in a Schema answered correctly by the learner out of the total number of MCQs attempted. A pie chart accompanies this to provide a visual representation. A score less than 50% is considered low and is denoted by a red title in the Schema list as shown above.

**Figure 3:**
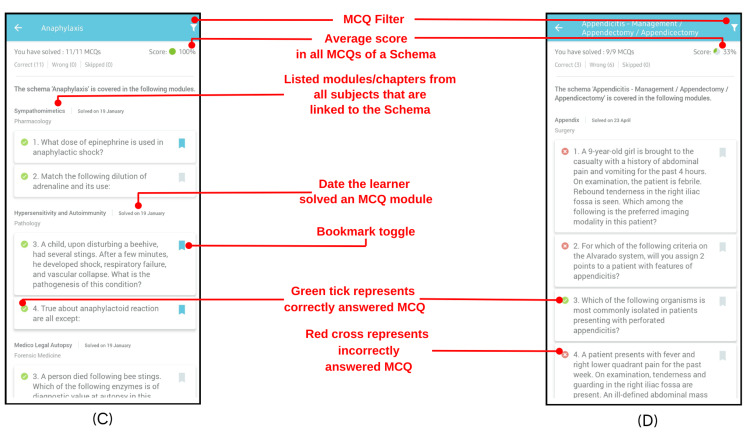
Schema showing the user's performance and granular feedback.

Green ticks and red crosses: Represent MCQs answered correctly and incorrectly, respectively. The learner can also filter either of these out with the help of the filter menu as shown in Figure 3.

Analysis of Performance and Trends in Medical Learning

It is possible to identify trends in the learning and performance of students by analyzing the pooled data of all learner groups on specific Schema topics. This can help locate learning gaps and assist in their closure through appropriate interventions. It can also offer insights into the necessity of focusing on specific competencies and changes in the learning landscape with changing illness burdens over time. We further explored this hypothesis through an in-depth retrospective analysis of anonymized Schema data. The granulated curricular organization of Schema, along with its capability to analyze pooled data, has the potential to observe, analyze, and augment the learning of cohorts of learners and the medical student community as a whole.

Unlike many learning tools and commercial MedEdTech platforms that primarily serve as content repositories or static test-preparation aids, Schema is designed as a dynamic, learner-centered ecosystem. It enables users to self-direct active learning, receive continuous actionable feedback, and engage with clinical reasoning through structured MCQs that test critical thinking in clinical scenarios and aids clinical decision-making skills and acumen. In this regard, Schema is a holistically designed system that combines the principles of quantified learning, ​​technology-enhanced learning (TEL), longitudinal learning, self-directed learning (SDL), PBL, simulation-based learning (SBL), feedback-directed learning, and integration across domains [8-10]. These modalities have been shown to outperform traditional teaching and learning models [11]. Furthermore, unlike traditional classroom learning, it allows learners to tailor their learning according to their unique requirements, directions, and personal competencies, thus creating a personalized learning experience. This holistic approach allows Schema to be integrated into the current undergraduate learning and assessment models to improve outcomes and quantify competencies. 

SOC-MCQs

Among the various components that constitute the Schema learning model, SOC-MCQs serve as a foundational tool for both learning and assessment. While SOC-MCQs are well-established for evaluating conceptual understanding and clinical reasoning, they inherently focus on structured problem-solving and may not fully capture communication skills, procedural proficiency, or nuanced decision-making. However, when embedded within a quantified, feedback-rich system like Schema, their effectiveness is significantly enhanced. Each MCQ in Schema is curated by a team of practicing doctors and is paired with detailed explanations that appear immediately after the learner submits a response. This pairing transforms SOC-MCQs from mere assessment tools into active learning stimuli that reinforce concepts and promote deeper cognitive engagement. Although SOC-MCQs alone have known limitations, their integration within Schema’s broader framework of feedback-driven, topic-wise progression and real-time performance tracking makes them a powerful tool for fostering self-directed, reflective learning [12].

Participants

We analyzed the anonymized data for 20 Schema topics, and the MCQs linked to them from 2018 to 2022 were shortlisted based on the relevance of knowledge in clinical practice. These topics included Anaphylaxis, ST-elevation myocardial infarction (STEMI) (STEMI), Covid-19 Vaccination, Glasgow Coma Scale, Subdural Hemorrhage, Syndromic Management of STIs, Laryngeal Mask Airway, BLS (Basic Life Support), Ischemic Stroke - Management, Ischemic Stroke - Risk Factors, Extradural Hemorrhage, NSTEMI, Acetaminophen, Management of Asthma, Diabetic Ketoacidosis (DKA), Hypertension - Management, Subarachnoid Hemorrhage, Vaccine Vial Monitoring, Vaccine & Drug Storage, and Community-Acquired Pneumonia - Management. The number of learners to attempt each MCQ ranged from 60,080 to 206,672.

Outcomes

A total of over 16,000 SOC-MCQs, spanning 19 core medical subjects, were non-exclusively mapped to 1,947 high-yield, subject-agnostic subtopics, which we refer to as Schema topics. Each MCQ could be linked to one or multiple Schema topics based on thematic and conceptual relevance. The number of MCQs assigned per topic varied between 1 and 47, depending on the complexity and curricular weightage of the subtopic. These topics were consolidated into a structured Schema interface that allowed users to intuitively navigate, filter, and track their progress across this granular curriculum. The interface provided real-time visual and quantitative feedback on topic-wise completion, accuracy, and performance trends. Schema was deployed as part of a mobile application available on both Android and iOS platforms and has been accessed by over 300,000 undergraduate medical students across the Indian subcontinent. Schema topics that showed statistically significant improvement, shown as an increase in percentage correctness over time, were primarily related to medical emergencies, including anaphylaxis, STEMI, Glasgow Coma Scale (36.73% improvement), acute subdural hemorrhage (5.29%), laryngeal mask airway devices (8.31%), and basic life support (BLS) (13.97%). This was determined through a retrospective, observational study design using anonymized performance data collected from users of the Schema app between 2018 and 2022.

The primary hypothesis was that Schema, as a quantified learning system, enables progressive improvement in learner competence over time for clinically important topics. For each Schema topic, we compared the average percent correctness of MCQ responses between two cohorts: an early cohort (2018-2019) and a recent cohort (2021-2022). An independent two-sample t-test was used to evaluate differences in mean performance across these cohorts. Statistical significance was considered at p < 0.001. The dataset comprised aggregated performance metrics from between 60,080 to 206,672 learner attempts per topic, which were obtained from the Schema platform’s backend analytics. The results support the hypothesis that quantified learning with timely feedback and topic-wise performance tracking can lead to measurable gains in competency, particularly in high-yield, time-sensitive clinical areas.

To assess Schema's capability for monitoring and improving competencies, we conducted a retrospective cohort analysis of anonymized performance data between 2018 and 2022. This analysis focused on 20 clinically relevant Schema topics. We observed that eight topics demonstrated statistically significant improvements in average MCQ correctness over time, as determined by an independent two-sample t-test (p < 0.001).

## Discussion

This innovation report describes Schema, a holistically designed, quantified e-learning system for undergraduate medical education. The retrospective analysis referenced in this report involved pooled, anonymized performance data from over 300,000 medical students across a four-year period (2018-2022), focusing on 20 high-yield Schema topics. It examined changes in learner competence, measured by percent correctness of SOC-MCQ responses, across different time cohorts. To our knowledge, this is the first large-scale attempt to collectively quantify medical learning outcomes across specific clinical competencies in real-world settings. The findings illustrate potential use cases of Schema in tracking evolving competency trends, identifying knowledge gaps, and informing both individual learning paths and broader curriculum development strategies. Similar trend analyses on a larger scale hold the potential to provide insights into the changing paradigms of competence and competencies over time, which in turn can guide targeted efforts toward the improvement of teaching-learning outcomes, both at an individual and an institutional level. On an individual level, Schema allows each learner to quantify their learning, assess their knowledge, learn actively, improve their competence with the help of active feedback, and practice clinical decision-making skills by practicing clinical scenarios. It acts as a seamless and holistic integration of the first principles of modern learning techniques such as self-directed learning, active recall, spaced repetition, feedback-based improvement, and curriculum integration, and in doing so, innovates the undergraduate learning landscape.

Technology has long been known to have the potential to transform learning [13]. Quantification has been the focus of many efforts in higher education and has sparked debate regarding the right approach to measuring outcomes in education [14]. Medicine remains a domain where true quantification of learning, assessment, and outcomes has yet to be explored, likely due to the many challenges unique to medicine, such as the vast curriculum, overlapping knowledge areas, complex learning patterns, and regulations [13].

Regarding the impact of Schema as a tool for self-directed learning, assessment, and improvement in medical education, evidence-based observations need to be collected using various applicable research methodologies. Most notably, it needs to be explored in light of the metrics provided by Messik in 1984, as well as the five markers of validity jointly prescribed by the American Educational Research Association (AERA), the American Psychological Association (APA), and the National Council on Measurement in Education (NCME) [15,16]. Avenues for integrating Schema into currently existing medical education models, consequences of such integrations, predictive models, and potential impacts of Schema need to be determined and aligned to the rapidly changing medical education landscape [17]. The impact itself may reach beyond learning outcomes. Various studies have remarked on the profound impact of better physician knowledge on patient outcomes [18-20]. Furthermore, Schema can be expanded to other levels of medical education, such as specialized and superspecialized courses, where granularization of the curriculum can be useful for in-depth assessment.

## Conclusions

Schema showcases vast potential to augment and disrupt the traditional ways we learn medicine. This first report paper demonstrates the different aspects of Schema and its applications. As we progress with the subsequent phases of understanding and developing Schema and its integrations, we plan to undertake further in-depth analysis and derive validity evidence. With this innovative solution, we aim to quantify learning, assessments, and improvements in academic outcomes for medical students and, by extension, hope to improve doctor-patient outcomes with a positive overarching impact on healthcare.

## References

[1] Eckleberry-Hunt J, Lick D, Hunt R (2018). Is medical education ready for generation Z?. J Grad Med Educ.

[2] Wallace S, Clark M, White J (2012). 'It's on my iPhone': attitudes to the use of mobile computing devices in medical education, a mixed-methods study. BMJ Open.

[3] Chase TJ, Julius A, Chandan JS (2018). Mobile learning in medicine: an evaluation of attitudes and behaviours of medical students. BMC Med Educ.

[4] Chandran VP, Balakrishnan A, Rashid M (2022). Mobile applications in medical education: a systematic review and meta-analysis. PLoS One.

[5] Sadler J, Wright J, Vincent T, Kurka T, Howlett D (2021). What is the impact of Apps in medical education? A study of CAPSULE, a case-based learning App. BMJ Simul Technol Enhanc Learn.

[6] Ščavnický J, Karolyi M, Růžičková P, Komenda M (2018). Pitfalls in users’ evaluation of algorithms for text-based similarity detection in medical education. 2018 Federated Conference on Computer Science and Information Systems.

[7] Karolyi M, Komenda M, Janoušová R, Víta M, Schwarz D (2016). Finding overlapping terms in medical and health care curriculum using text mining methods: rehabilitation representation - a proof of concept. Mefanet J.

[8] Melo J, Kaneshiro B, Kellett L, Hiraoka M (2014). The impact of a longitudinal curriculum on medical student obstetrics and gynecology clinical training. Hawaii J Med Public Health.

[9] Ellaway RH (2018). Technology-enhanced learning. Understanding Medical Education: Evidence, Theory, and Practice, Third Edition.

[10] Atta IS, Alghamdi AH (2018). The efficacy of self-directed learning versus problem-based learning for teaching and learning ophthalmology: a comparative study. Adv Med Educ Pract.

[11] Zahid MA, Varghese R, Mohammed AM, Ayed AK (2016). Comparison of the problem based learning-driven with the traditional didactic-lecture-based curricula. Int J Med Educ.

[12] Mehta M, Banode S, Adwal S (2016). Analysis of multiple choice questions (MCQ): important part of assessment of medical students. Int J Med Res Rev.

[13] Fuller R, Goddard VC, Nadarajah VD (2022). Technology enhanced assessment: Ottawa consensus statement and recommendations. Med Teach.

[14] Kandiko HC, Buckley A (2020). Quantifying learning: measuring student outcomes in higher education in England. Polit Gov.

[15] Messick S (1984). The psychology of educational measurement. J Educat Measurement.

[16] American Educational Research Association (2014). American Psychological Association, National Council on Measurement in Education, Joint Committee on Standards for Educational and Psychological Testing (U.S.): Standards for Educational and Psychological Testing. https://www.apa.org/science/programs/testing/standards.

[17] de Bruin AB, Dunlosky J, Cavalcanti RB (2017). Monitoring and regulation of learning in medical education: the need for predictive cues. Med Educ.

[18] Clark NM, Gong M, Schork MA (1998). Impact of education for physicians on patient outcomes. Pediatrics.

[19] Gaupp R, Körner M, Fabry G (2016). Effects of a case-based interactive e-learning course on knowledge and attitudes about patient safety: a quasi-experimental study with third-year medical students. BMC Med Educ.

[20] Choy CL, Liaw SY, Goh EL, See KC, Chua WL (2022). Impact of sepsis education for healthcare professionals and students on learning and patient outcomes: a systematic review. J Hosp Infect.

